# Supplementary material for: Genome dependent Cas9/gRNA search time underlies sequence dependent gRNA activity
Source: Nat Commun. 2021 Aug 19;12:5034. doi: 10.1038/s41467-021-25339-3 (PMC8377084; doi:10.1038/s41467-021-25339-3)
Supplement: Supplementary file 1 — Supplementary Information [file 41467_2021_25339_MOESM1_ESM.pdf]

Genome Dependent Cas9/gRNA Search Time Underlies Sequence Dependent gRNA Activity.

Moreb, E.A[1], and Lynch, M.D.[1,2,3]

[1]Department of Biomedical Engineering, Duke University

[2] To whom all correspondence should be addressed.

[3] michael.lynch@duke.edu

Supplementary Materials

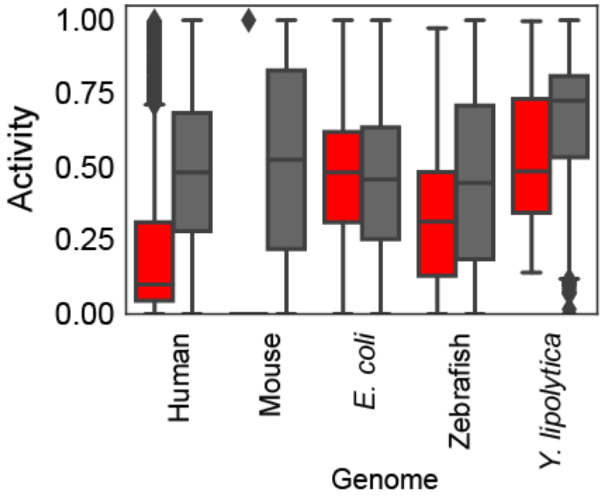

Supplementary Figure S1: Boxplots of gRNA activity containing four thymines (red) anywhere in the 20 bp targeting portion of the gRNA vs average activity of gRNA without four thymines (grey). From left to right, n=8,672; n=326,774; n=7; n=1,149; n=10,290; n=120,459; n=46; n=1,168; n=1,808; n=44,412.

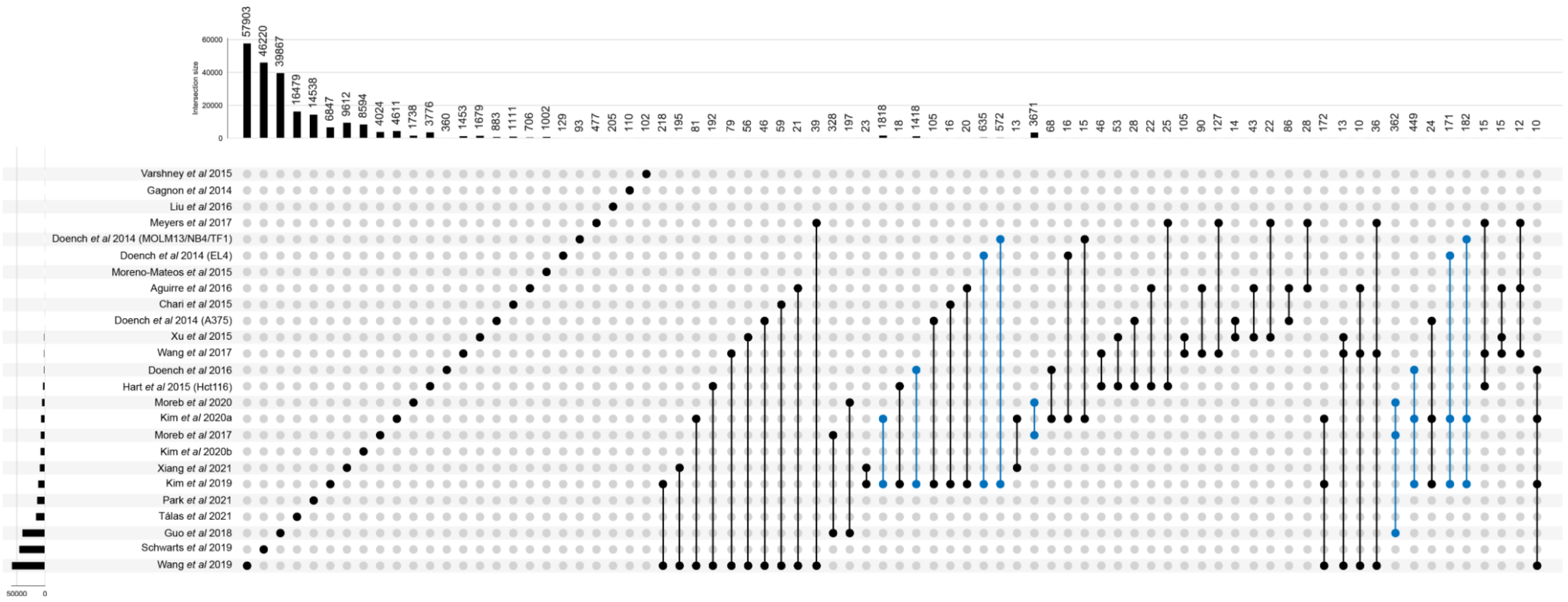

Supplementary Figure S2: The number of gRNA shared between datasets. The bar graph at the top of the plot shows the number of gRNA that are present in the dataset(s) indicated. A single point indicates that the gRNA are unique to that specific dataset while a line connecting two or more points indicates that the gRNA are unique to those shared datasets. All bars with 10 or fewer gRNA have been excluded from this plot. Blue connections show overlapping gRNA that were excluded from Figure 5 in one of the connected datasets.

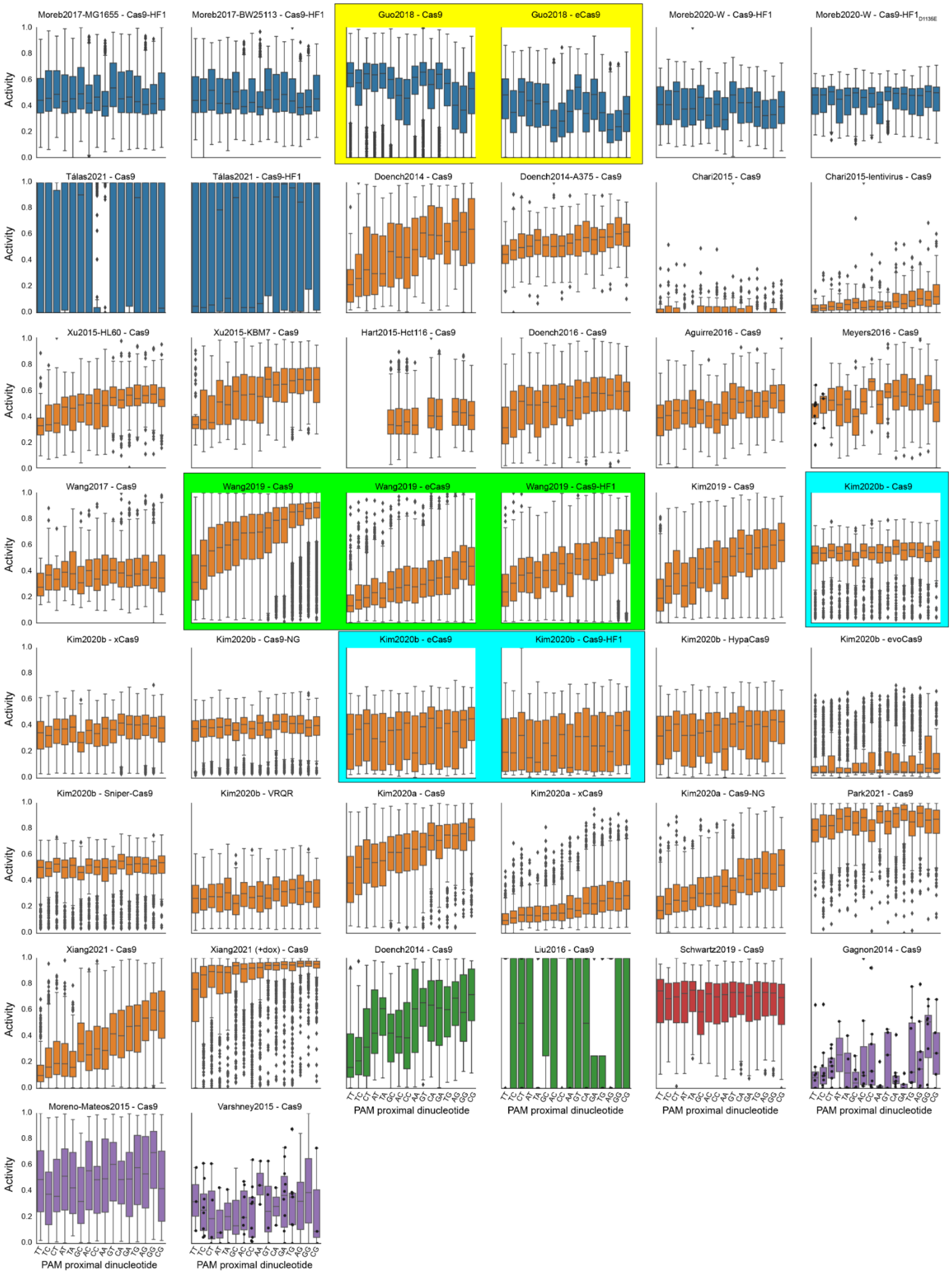

Supplementary Figure S3: Activity of gRNA grouped by PAM proximal dinucleotide sequence. Plots represent the data in the pairwise correlations in Figure 5 in the main text. The dinucleotide sequences are ordered by least active to most active based on the average activity per dinucleotide in the largest human dataset, from Wang *et al* 2019[1]. Each box-and-whisker plot represents the distribution of gRNA activities that start with that specific dinucleotide sequence. The bars extend to 1.5 x (the interquartile range) and

gRNA outside of this range are represented as diamonds. In cases where $n \leq 10$, black dots have been overlaid on the boxplots. Colors of boxes represent different species: Blue = *E. coli*; Orange = Human; Green = Mouse; Red = *Y. lipolytica*; Purple = Zebrafish. Additionally, for datasets where Cas9, Cas9-HF1, and/or eCas9 are reported together, we highlight that activity grouped by the PAM proximal dinucleotides does not significantly change. These data suggest that context specific impact on the PAM proximal sequence is consistent across these Cas9 variants in data from Guo *et al* 2018[2] (yellow shading), Wang *et al* 2019[1] (green shading), and Kim *et al* 2020b[3] (teal shading).

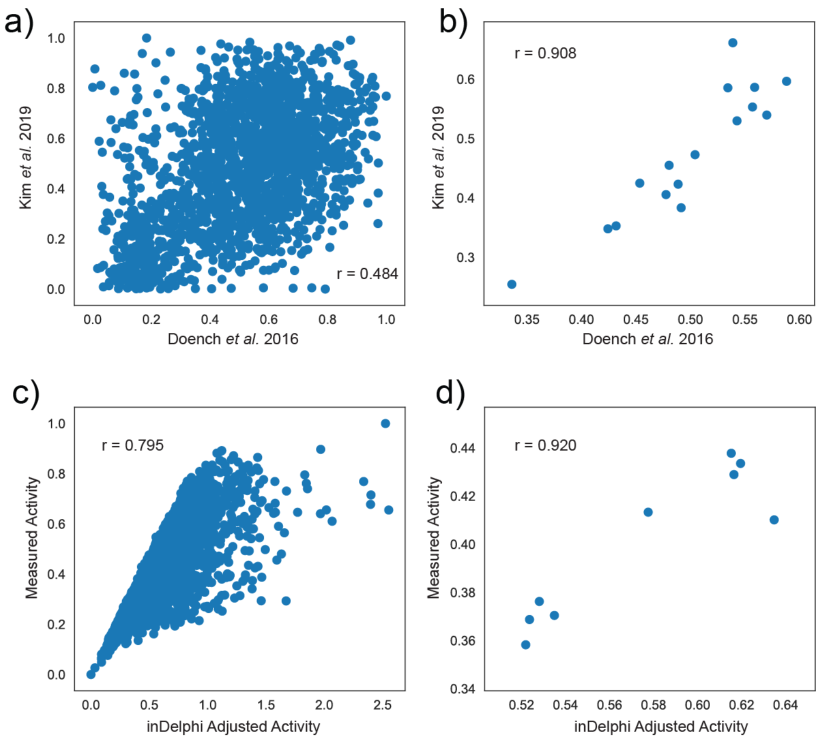

Supplementary Figure S4: Assessing the impact of gRNA screening method on PAM proximal sequence-based averages. One of the challenges in assessing gRNA activity in phenotypic screens is that the indels generated do not always lead to gene knock-outs. Since indel formation is somewhat predictable and sequence dependent, we wanted to assess how differences in measuring gRNA activity might influence the PAM proximal sequence preference. 1,899 gRNA from Doench *et al* 2016[5] were included in the library screened by Kim *et al* 2019[6]. The Doench et al screen used a phenotypic readout of activity while Kim *et al* used a more direct sequencing based readout. a) The measured activity for each gRNA is modestly correlated (r=0.484) between the two screens. b) However, if we group gRNA by the PAM proximal 2 base pairs and calculate the average activity per group, we see a strong correlation between these averaged values (r=0.908). This suggests that the relative impact of the PAM proximal sequence is the same within each dataset, despite the differences in how the screens measure activity. To further assess this, we used inDelphi to calculate an "inDelphi Adjusted Activity" score for the dataset from Hart *et al* 2015.[7,8] This dataset screen was performed in cell line Hct116, one of the cell lines included in the inDelphi model. inDelphi provides a predicted "Frameshift Frequency" for a given DNA break based on the surrounding nucleotides. Given that measured activity in a phenotypic screen would only capture frameshifts which lead to knock-outs, we reasoned that: Actual Activity x Frameshift Frequency = Measured Activity. c) Using this logic, we calculated what the actual activity should be ("inDelphi Adjusted Activity") and compared it to the measured activity for each gRNA. We found a strong correlation (r=0.795) between measured Activity and inDelphi Adjust Activity, suggesting that phenotypic screens are mostly capturing gRNA activity accurately. d) Furthermore, we found that when calculating the average activity by the PAM proximal 2bp, the averaged inDelphi Adjusted Activity was strongly correlated with the averaged Measured Activity. This further confirms the strong correlations between PAM proximal sequence-based averages in human datasets that we see in Figure 5, despite differences in screening methods.

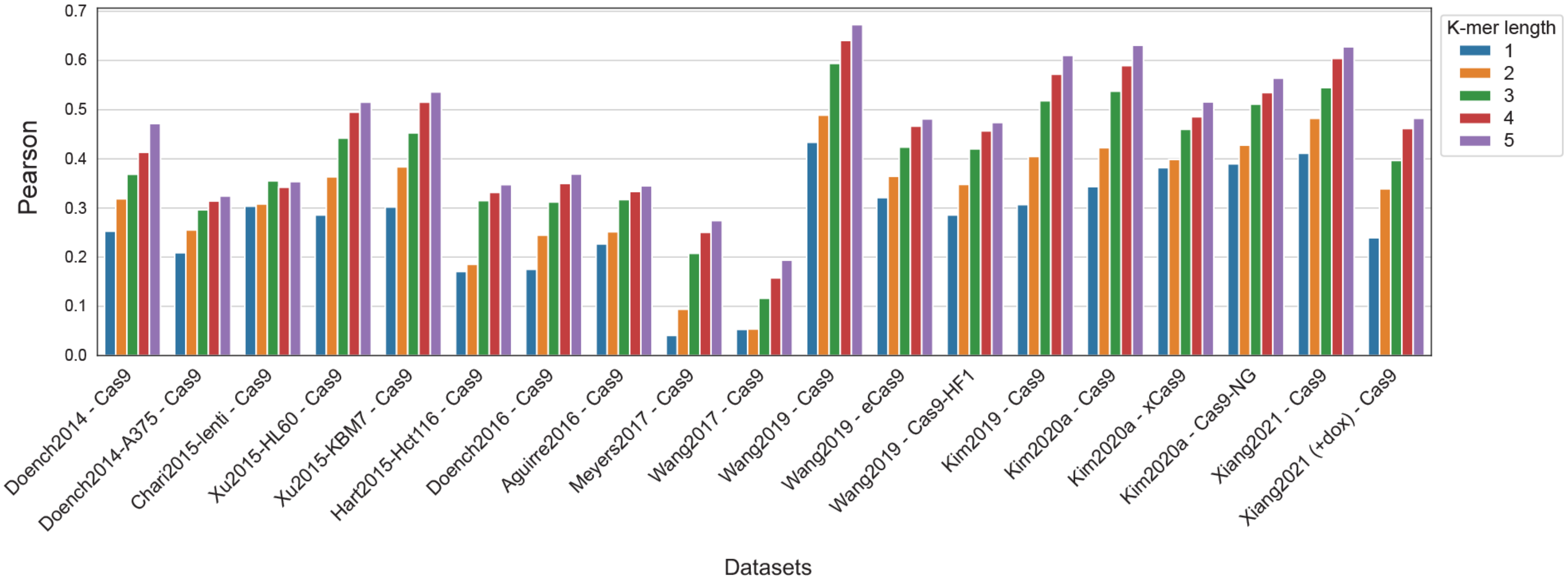

Supplementary Figure S5: Predictions within human datasets using the PAM proximal sequence based activity averages (see Figure 7), improve with longer K-mer sequence. Excluded datasets with modified gRNA scaffold and the Chari *et al* 2015[9] dataset targeting endogenous sites, due to the overall low activity of that dataset. n=1 for a single prediction.

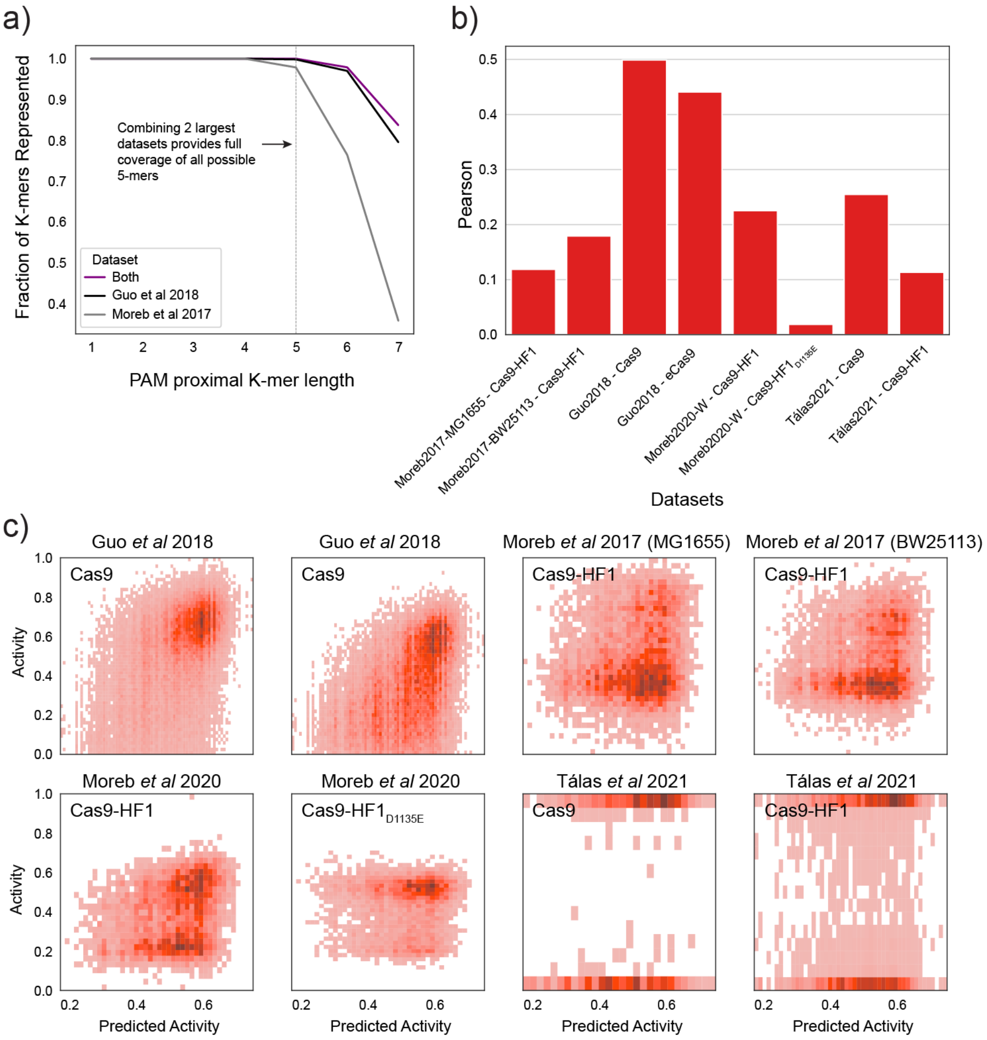

Supplementary Figure S6: Using a similar approach as that of Figure 7 in the main text, we combined data from Guo *et al* 2018[2] and Moreb *et al* 2017[10] to produce predictions based on the PAM proximal 5 bp sequence. a) Combining these two datasets gives full coverage of all 5-mer sequences. b) Predictions based on the PAM proximal sequence are evaluated based on Pearson correlation with actual activity. c) For each dataset, we plot actual activity against predicted activity.

Supplementary Note 1:

Proposed gRNA library guidelines for understanding context specific sequence preferences in new species

In this analysis, we highlighted that the PAM proximal sequence is most correlated with gRNA activity in a host specific manner, therefore it is important for predicting the activity of given gRNA within a specific context. In Supplementary Figure S5, we show that increasing the length of the PAM proximal sequence used for predictions improves the predictive power of this approach. However, no datasets have yet been designed to systematically evaluate context dependent sequence-preference in this manner. For understanding context-dependent gRNA activity in new hosts, it may therefore be beneficial to systematically assess context dependent factors. Here we highlight factors to consider while designing a gRNA library in novel host contexts:

1. To systematically assess the impact of context, it may be beneficial to design sequence diversity in the PAM proximal sequence by selecting *n* replicates of each K-mer, where K represents the PAM proximal sequence length. In general, larger *n* and longer K will provide a better understanding of context. However, selecting the appropriate *n* and K values is dependent on practical variables, such as the throughput of the gRNA screen, budget, and constraints. To help researchers select the appropriate *n* and K for their gRNA library design, we calculated the library size with different *n* and K values (Supplementary Figure S7a), as well as the potential predictive ability of a library designed with different *n* and K values (Supplementary Figure S7b).
2. In addition to designing variability within the PAM proximal sequence, researchers may also want to account for unwanted secondary structure within the gRNA,[11,12] Cas9 preference for NGGH PAM sites,[13] and species-specific transcriptional modifiers (ie, four thymines in human or mouse cell lines[14]).

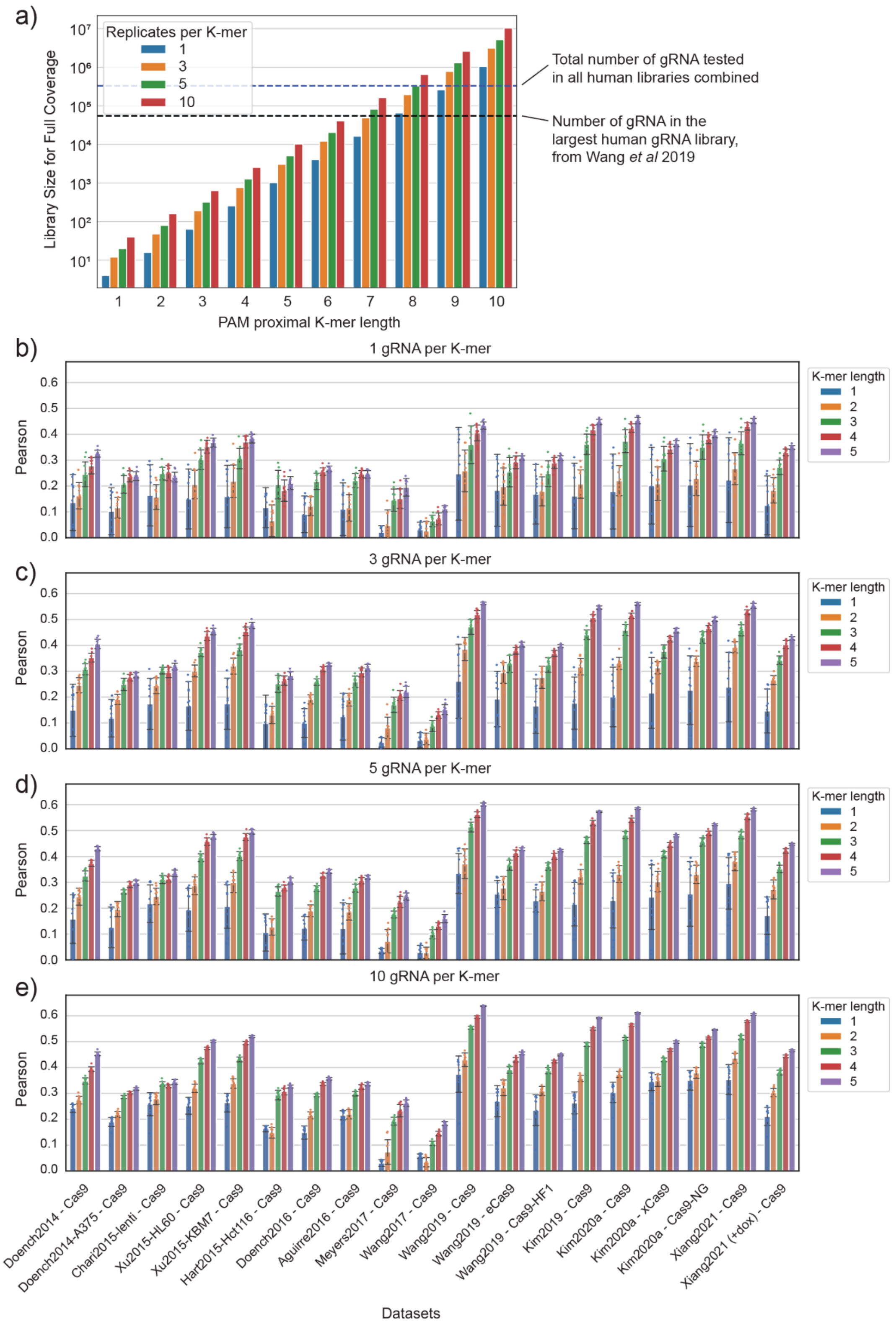

Supplementary Figure S7: a) We calculate the required library size to provide complete coverage for all PAM proximal K-mer sequences (K between 1 and 10 base pairs) and for different numbers of replicates per unique K-mer. For example, to get complete coverage of all possible 5-mer PAM proximal sequences

with 3 replicates per 5-mer, you would need a library size of just 3,072 gRNA. Provided as reference are the total number of gRNA in all the human datasets collected in this paper (black dashed line) and the number of gRNA in the largest dataset in this paper (blue dashed line). Following the steps of the analysis shown in Figure 7, we combined the wild-type Cas9 datasets from Wang *et al* 2019[1] and Kim *et al* 2019[6] and randomly sampled either b) 1, c) 3, d) 5, or e) 10 gRNA per K-mer to look at the number of replicates required to evaluate context specific differences in host context. After random sampling, we grouped gRNA by the PAM proximal K-mer sequence and used the averages to predict activity in other human datasets using the original gRNA scaffold. We repeated the sampling and prediction steps n=10 times and plot the average of these 10 predictions. The bars represent the standard deviation and the dots are the individual data points.

References

1. Wang, D. *et al.* Optimized CRISPR guide RNA design for two high-fidelity Cas9 variants by deep learning. *Nat. Commun.* **10**, 4284 (2019).
2. Guo, J. *et al.* Improved sgRNA design in bacteria via genome-wide activity profiling. *Nucleic Acids Res.* **46**, 7052–7069 (2018).
3. Kim, N. *et al.* Prediction of the sequence-specific cleavage activity of Cas9 variants. *Nat. Biotechnol.* **38**, 1328–1336 (2020).
4. Kim, H. K. *et al.* High-throughput analysis of the activities of xCas9, SpCas9-NG and SpCas9 at matched and mismatched target sequences in human cells. *Nat Biomed Eng* **4**, 111–124 (2020).
5. Doench, J. G. *et al.* Optimized sgRNA design to maximize activity and minimize off-target effects of CRISPR-Cas9. *Nat. Biotechnol.* **34**, 184–191 (2016).
6. Kim, H. K. *et al.* SpCas9 activity prediction by DeepSpCas9, a deep learning-based model with high generalization performance. *Sci Adv* **5**, eaax9249 (2019).
7. Shen, M. W. *et al.* Predictable and precise template-free CRISPR editing of pathogenic variants. *Nature* **563**, 646–651 (2018).
8. Hart, T. *et al.* High-Resolution CRISPR Screens Reveal Fitness Genes and Genotype-Specific

Cancer Liabilities. *Cell* **163**, 1515–1526 (2015).

9. Chari, R., Mali, P., Moosburner, M. & Church, G. M. Unraveling CRISPR-Cas9 genome engineering parameters via a library-on-library approach. *Nat. Methods* **12**, 823–826 (2015).
10. Moreb, E. A. *et al.* Managing the SOS Response for Enhanced CRISPR-Cas-Based Recombineering in E. coli through Transient Inhibition of Host RecA Activity. *ACS Synth. Biol.* **6**, 2209–2218 (2017).
11. Wong, N., Liu, W. & Wang, X. WU-CRISPR: characteristics of functional guide RNAs for the CRISPR/Cas9 system. *Genome Biol.* **16**, 218 (2015).
12. Tálas, A. *et al.* A method for characterizing Cas9 variants via a one-million target sequence library of self-targeting sgRNAs. *Nucleic Acids Res.* (2021) doi:10.1093/nar/gkaa1220.
13. Doench, J. G. *et al.* Rational design of highly active sgRNAs for CRISPR-Cas9-mediated gene inactivation. *Nat. Biotechnol.* **32**, 1262–1267 (2014).
14. Hsu, P. D. *et al.* DNA targeting specificity of RNA-guided Cas9 nucleases. *Nat. Biotechnol.* **31**, 827–832 (2013).
