# Supplementary material for: Genome dependent Cas9/gRNA search time underlies sequence dependent gRNA activity
Source: Nat Commun. 2021 Aug 19;12:5034. doi: 10.1038/s41467-021-25339-3 (PMC8377084; doi:10.1038/s41467-021-25339-3)
Supplement: Supplementary file 10 — Reporting Summary [file 41467_2021_25339_MOESM10_ESM.pdf]

nature research

Corresponding author(s): Michael Lynch

Last updated by author(s): Aug 2, 2021

Reporting Summary

Nature Research wishes to improve the reproducibility of the work that we publish. This form provides structure for consistency and transparency in reporting. For further information on Nature Research policies, see our Editorial Policies and the Editorial Policy Checklist.

Statistics

For all statistical analyses, confirm that the following items are present in the figure legend, table legend, main text, or Methods section.

n/a	Confirmed	
☐	☒	The exact sample size (n) for each experimental group/condition, given as a discrete number and unit of measurement
☐	☒	A statement on whether measurements were taken from distinct samples or whether the same sample was measured repeatedly
☐	☒	The statistical test(s) used AND whether they are one- or two-sided<br>*Only common tests should be described solely by name; describe more complex techniques in the Methods section.*
☐	☒	A description of all covariates tested
☐	☒	A description of any assumptions or corrections, such as tests of normality and adjustment for multiple comparisons
☐	☒	A full description of the statistical parameters including central tendency (e.g. means) or other basic estimates (e.g. regression coefficient) AND variation (e.g. standard deviation) or associated estimates of uncertainty (e.g. confidence intervals)
☐	☒	For null hypothesis testing, the test statistic (e.g. F, t, r) with confidence intervals, effect sizes, degrees of freedom and P value noted<br>*Give P values as exact values whenever suitable.*
☒	☐	For Bayesian analysis, information on the choice of priors and Markov chain Monte Carlo settings
☒	☐	For hierarchical and complex designs, identification of the appropriate level for tests and full reporting of outcomes
☐	☒	Estimates of effect sizes (e.g. Cohen's d, Pearson's r), indicating how they were calculated

Our web collection on statistics for biologists contains articles on many of the points above.

Software and code

Policy information about availability of computer code

Data collection	No software was used to collect data.
Data analysis	All computation was performed in Python version 3.8. Datasets were managed with Pandas version 1.0.1. NumPy version 1.18.1 was used for calculations. Regex was used for finding gRNA sequences in reference genomes. Scipy version 1.4.1 was used for statistics and scikit-learn version 0.22.2.post1 was used for linear regressions. Seaborn version 0.11.1 and Matplotlib version 3.1.3 were used for plotting. Biopython version 1.76 was used for calculating melting temperatures. Folding energies of gRNA were calculated using ViennaRNA RNAfold package version 2.4.17. Code was written in Jupyter Notebook version 6.0.3 and is provided as a Supplemental File.

For manuscripts utilizing custom algorithms or software that are central to the research but not yet described in published literature, software must be made available to editors and reviewers. We strongly encourage code deposition in a community repository (e.g. GitHub). See the Nature Research guidelines for submitting code & software for further information.

Data

Policy information about availability of data

All manuscripts must include a data availability statement. This statement should provide the following information, where applicable:

- Accession codes, unique identifiers, or web links for publicly available datasets
- A list of figures that have associated raw data
- A description of any restrictions on data availability

All data collected and used in this manuscript are freely available and attached as Supplemental Data. The code written for all analysis is included as a Jupyter Notebook file in Supplemental File 1.

Field-specific reporting

Please select the one below that is the best fit for your research. If you are not sure, read the appropriate sections before making your selection.

[x] Life sciences [ ] Behavioural & social sciences [ ] Ecological, evolutionary & environmental sciences

For a reference copy of the document with all sections, see nature.com/documents/nr-reporting-summary-flat.pdf

Life sciences study design

All studies must disclose on these points even when the disclosure is negative.

Sample size	Data was collected from the literature. We did not determine the sample size.
Data exclusions	As described in Methods, gRNA targeting the host genome were excluded if they could not align to the genome. As outlined in methods and figure captions, we removed subsets of overlapping gRNA for figures 5 and 6.
Replication	We did not reproduce any experimental work.
Randomization	For model building, we randomly split the data into 80% training groups and 20% testing groups for each dataset. Each randomization and training was repeated 10 times.
Blinding	We did not perform or repeat experimental studies and so did not blind. For model building, training and testing groups were assigned randomly via computation and therefore inherently blinded.

Reporting for specific materials, systems and methods

We require information from authors about some types of materials, experimental systems and methods used in many studies. Here, indicate whether each material, system or method listed is relevant to your study. If you are not sure if a list item applies to your research, read the appropriate section before selecting a response.

Materials & experimental systems

n/a	Involved in the study	
[x]	[ ]	Antibodies
[x]	[ ]	Eukaryotic cell lines
[x]	[ ]	Palaeontology and archaeology
[x]	[ ]	Animals and other organisms
[x]	[ ]	Human research participants
[x]	[ ]	Clinical data
[x]	[ ]	Dual use research of concern

Methods

n/a	Involved in the study	
[x]	[ ]	ChIP-seq
[x]	[ ]	Flow cytometry
[x]	[ ]	MRI-based neuroimaging
