# Supplementary material for: Genome dependent Cas9/gRNA search time underlies sequence dependent gRNA activity
Source: Nat Commun. 2021 Aug 19;12:5034. doi: 10.1038/s41467-021-25339-3 (PMC8377084; doi:10.1038/s41467-021-25339-3)
Supplement: Supplementary file 11 — Description of Additional Supplementary Files [file 41467_2021_25339_MOESM11_ESM.pdf]

Title: Supplementary Data 1:
Description: Provides an overview of each dataset used in this paper, including reference information and dataset features used in Figure 2.

Title: Supplementary Data 2:
Description: Contains gRNA sequence, activity and other features for gRNA libraries evaluated in human cell lines.

Title: Supplementary Data 3:
Description: Contains gRNA sequence, activity and other features for gRNA libraries evaluated in E. coli.

Title: Supplementary Data 4:
Description: Contains gRNA sequence, activity and other features for gRNA libraries evaluated in mouse cell lines.

Title: Supplementary Data 5:
Description: Contains gRNA sequence, activity and other features for gRNA libraries evaluated in Y. lipolytica.

Title: Supplementary Data 6:
Description: Contains gRNA sequence, activity and other features for gRNA libraries evaluated in zebrafish.

Title: Supplementary Data 7:
Description: Contains gRNA sequence, activity and other features for gRNA libraries evaluated in human cell lines using nuclease null Cas9 variants: dCas9 and base editors.

Title: Supplementary Software
Description: Python code containing all analysis is included as a Jupyter Notebook
